# Supplementary material for: Development and validation of School Oral Health Promotion Program “SOHEPP” oral health video for the training of adolescents and teachers in Ibadan, Nigeria
Source: PLOS Glob Public Health. 2025 Apr 22;5(4):e0004521. doi: 10.1371/journal.pgph.0004521 (PMC12013892; doi:10.1371/journal.pgph.0004521)
Supplement: S1 Text — (DOCX) [file pgph.0004521.s001.docx]

**Transcripts**

**WhatsApp and Phone calls**

**Teacher One**

I have taken time to watch the video sent. My comments: the video was well composed and arranged, presentation cuts across ages and the message was clearly explained. It is highly educative and informative. To have widest coverage of audience, do you permit it being forwarded to various platforms and groups one belongs to? Also, if a step can be taken further to translate the message into three common Nigerian languages. This will enhance widest dissemination of the message. Schools with projectors can even project it on a screen to make the message permanent in the students’ memories. From our school end here, it will be posted on the school’s official WhatsApp. Thank you.

**Teacher Two**

The video is presentable for everyone as most of the information needed are there. The causes of the tooth problems were well explained there. The information can be developed for less-literate individuals for them to know the tooth problems and how they can go about it instead of using battery water.

**Teacher Three**

Yes, it is appropriate for both the teachers and students.

It is well explanatory and super educative.

None from my end (what should be added).

The message of the video is well passed across. It is enlightening.

**Teacher Four**

Yes, it is appropriate for students and teachers.

It educates and informs people about caring for their teeth and mouth.

I will love it if it can be performed in other languages e.g. Advanced English, Yoruba, Hausa, etc. The contents of the video is incredible.

**Teacher Five**

I have no additions or subtraction. The video is okay.

**Teacher Six**

I have no comments. It was professionally done.

**Teacher Seven**

I have no comments. Thank you. Well done.

**Teacher Eight**

No comment. Well done.

**Students**

**AFTER WATCHING THE VIDEO**

**SCHOOL ONE**

**PREAMBLE:** Introduction of the team and the students, explanation of the purpose of the meeting, giving of numbers to the students and obtaining permission to record the discussions.
**Interviewer:** Um now what are your views about the video you just watched? Do you still remember your number? What have you learnt from the video?

Respondent: I am number 3, I learnt that when we brush; we should brush, you brush: there are steps to take when brushing, we should avoid sugary foods and unbalanced diet.

**Interviewer:** Thank you.

Respondent: I am number 4, in the video they showed a dialogue program which um…showed us the proper hygienic way of taking care of our teeth, brush it every day.

Respondent: I am number 6, in the video they showed us the amount of toothpaste we are to use, some people spread it on the brush, and it is supposed to be pea size.

Respondent: I also learnt that if we want to brush our teeth, we should be extra careful when brushing it to avoid blood coming out from the gum; it can cause some disease that and maybe we are eating very hard foods, we should remove it so that it won’t decay afterwards.

**Interviewer:** With what?

Respondent: flossing.

**Interviewer:** Dental floss.
Respondent: I am number one. I learnt that after we drink anything that has acid, it should be 30 minutes before we brush our teeth.

**Interviewer:** Thank you. Now is the video appropriate? Is it ok for your age group? Yes, or no?

Respondents: Yes *(chorused).*

**Interviewer:** So why do you think is appropriate for your age?

Respondent: Number 2, Yes, it is okay for us. It is perfect for our age group because some of us do not know how to brush our teeth, so, it teaches us more on how to brush and be careful with our gum.

Respondent: Number 7, it is appropriate for our age because of the language they used, they used a kind of pidgin language and some teenage words.

Respondent: I am number 10, it is appropriate for us because it is in the form of um… animation, it is a form that is attractive for us to watch, we want to know everything that is going to happen because of the cartoon - it is not real human beings so I think that is within our age as we still watch all those cartoons.

**Interviewer:** What are those things that you personally like in that video?

Respondent: I am number 3, When they are talking about some diseases, which young people don't know about, like periodontal diseases and which me I don’t know, just enlightening or expatiating more on those diseases.

**Interviewer**: Thank you. So what are those things that you like about the video?

Respondent: I am number 10, what I like most is about the way you talk; I like the language that they used, like it is even making me more entertained.

Respondent: I am number 6, the thing I like about the video is when Dr. Fresh and the presenter were talking, the way he was interacting with the doctor.

Respondent: The way the doctor explained everything about the mouth odor and the diseases.

**Interviewer:** Thank you for that. Now the song at the end of that video do you like it?

Respondents: Yes

**Interviewer:** Can you sing the song? Okay let us try.

Respondents: …students sing… Oh, I was in that brush. I go brush…..
**Interviewer**: So, you like the song at the end of the video. What does that song teach you?

Respondent: I am number 4, that song was like a motivation to us. It was was like every morning you wake up you must brush.

**Interviewer**: Thank you. Now there are some things that you may think, oh, I don't like this video. Can you pinpoint those areas?

Respondent: I am number 10, the area I don’t like is where they showed us the teeth that decay, rotten teeth. It is irritating.

Respondent: Like the starting point, you know, it is not something so attractive, they should make it in a way that more, like, more comprehensive or more like on a lighter note.

**Interviewer:** Now you are taking me to the next question, that, how do you think we can make this video better? What are those things that you think we should add to make it better, to make a more interesting, more educative video, what do you think we should do?

Respondent: I am number 6, I want them to make it a more practical session that we can go home and practice.

Respondent: Okay I am number 10. I think the producer of the video should make a short video of some people on how to brush because not all people can brush like this way, so they should post it online for people to know the exact way to brush your teeth. Don't brush it like this, so the way this man….there was this man that was brushing his teeth, not everybody can do it like that and some people, their tooth is paining them. So, I think they should organize more video for that.

**Interviewer**: Thank you.

Respondent: And I think that in the video they are supposed to put a special Maclean or what can be used….…and the kind of toothbrush; some people will use hard, soft; they should make us understand the type of toothbrush and we should not be changing it on daily basis.

**Interviewer**: The next question is that, would you prefer this oral video in form of cartoon or drama, or which of them would you prefer? Or do you like it as it is, in form of this cartoon?

Respondent: For young children, this cartoon is okay but for adult… the human form is the best for them.

**Interviewer:** But for your age, is it okay?

Respondent: We are in the adulthood stage, so I think the human form is the best for us.

Respondent: It is okay like this, but it is more cartoonish, it should be a little bit adult-like, small children will like it like this but…

Interviewer: Will you prefer home theatre drama? Do you want it in form of drama or you just like it as it is?

Respondent: I think I like the dialogue; the dentist and the presenter should be the form of drama while other parts should be in cartoon.
**Interviewer**: Will you prefer this video to be longer in length or shorter?
Do you think we should shorten it, or we should make it longer?

Respondent: I think it is okay because in as much as it contains all the appropriate information we need to know about the teeth.
Respondent: It is not too long and not too short because it has all the necessary information, so there is no need to add any more content and there is no need to cut things out of it, so it is okay like this.
**Interviewer:** Thank you. What about the graphics, the pictures, and the brightness of the video, is it okay? What can you say about that?

Respondent: I am number 4, I would say that the graphics is okay I can say that it is in MP3 format and easy for all. I like that the animation, the way they were moving, it should correspond with what they were saying. At times the movement of the mouth did not correspond with what they were saying and we want it to be more advanced.

**Interviewer:** The graphics, the picture, the entire video, the brightness, is it okay by you?

Respondent: The brightness, I am number 1, the brightness is okay but for the graphics was like of low quality because they were just moving like slowly and it was very dull.

**Interviewer:** Does that mean it is boring to you?

Respondent: It is not boring.

**Interviewer**: Is it boring?

Respondents: No
**Interviewer:** Is it interesting?

Respondent: Yes.

**Interviewer**: Thank you. Now how do you think we can share this video with students and your teachers? How do you think we can go about that?

Respondent: You can share it to everybody online, not even us alone, because everybody is online now so if we get it online……

Respondents: Murmuring…..

**Interviewer:** So, somebody is saying here that some people have no access to online platforms, how do we deal with that? We want everybody to access the video.

Respondent: You can put it on TV shows, so many people watch TV than online.

Respondent: I am number five, those people that are not online, I am very sure that they will have somebody, someone close to them will be online, that I am very sure, even a two-year-old baby watch what is online now so everyone knows.

Respondent: After posting online, maybe you can paste it on billboards around, so that people that are, you know, walking, driving or riding in cars or …you always look at this, people especially me, I love doing that.

**Interviewer:** Thank you so much. Now in summary, do you like the video?

Respondents: Yes

**Interviewer:** Is the video interesting?

Respondents: Yes
**Interviewer**: Can you recommend the video to anybody, everybody?

Respondent: Yes

**Interviewer**: Any other opinion about the video before we end the discussion?

Respondent: *(Silence)*.

**Interviewer:** Thank you so much. I really thank you for being part of this focus group discussion, thank you so much.

**SCHOOL TWO**

**PREAMBLE:** Introduction of the team and the students, explanation of the purpose of the meeting, giving of numbers to the students and obtaining permission to record the discussions.
**Interviewer:** Now, what are your views about this video that you just watched? What do you think about the video? You tell me your number. Do you still remember your number?

Respondent: Number 5. How to protect our teeth, what causes the damage or disease, wash our tooth and then we should wash our tooth.

**Interviewer**: What do you… what have you learnt from that video?

Respondent: Number 1, I learnt the way we can protect our teeth from getting spoilt, what we should avoid to prevent our teeth from spoiling and the procedures of brushing and what spoils our teeth, what we should do and what we should not do.

Respondent: Number 2, I learnt that we should prevent our teeth from damage; we should care for our teeth and be eating diet that can help our teeth.

Respondent: I learnt that baby/child has 20 teeth called temporary teeth and adult has 32 teeth, which are called permanent teeth and I also learnt how to take care of our teeth and what causes damage to our teeth.

**Interviewer:** What did you learn? What's your opinion about that video?

Respondent: To brush our teeth two times a day.

Respondent: It teaches us not to be smoking.

Respondent: Regular visit to the dentist, I am number 13. We should eat fruits and vegetables and we should not smoke.

**Interviewer:** What did you learn? What's your opinion about that video?

Respondent: Silence.

**Interviewer:** The video, is it appropriate for your age, for your age group?
Do you think it's appropriate/good for your age group?

Respondents: Yes.

**Interviewer**: Why do you think it is good/appropriate for your age group?

Respondent: I think it is good for our age group because of, like, our community, the language they speak is pidgin and, like, related to our community, mostly and the graphic is like, nice cartoon, it also helps us to understand.

**Interviewer**: Okay?

Respondent: It teaches us many things. It makes me know that adults have 32 teeth and some parts of our teeth.

**Interviewer**: So, it is okay for your age group?

Respondent: It is good because it teaches us how to take care of our teeth

**Interviewer**: Okay, any other thing that you learned?

Respondent: Number 11, after eating in the morning or in the night, we should brush after eating a dinner, breakfast, lunch, we should brush at least three, two times daily, and we can brush for three minutes.

**Interviewer**: Okay, can you tell me any other thing that you learnt?

Respondent: *(Silence)*.

**Interviewer**: Now in that video, what are those things that you like in that video?
Respondent: I like how they taught us how to take care of our teeth and how to maintain our teeth.

**Interviewer:** So, what are those things? Okay, okay. That's what you like.

Respondent: Number 5, I like the music.

**Interviewer**: Okay. You like the music. Okay. What do you like?

Respondent: Number 1, in the movie, in the video, I like that they use teenagers, because the teenagers express themselves well and the pictures they used when they were explaining the way of treating the teeth and disease so that was like, those are what I liked.

**Interviewer:** Now, um, do you like the song at the end of the video?

Respondent: Yes.

**Interviewer**: Can you remember this song? Can you sing it?

Respondent: I don't.

**Interviewer:** You don't remember, you don't like it?

Respondent: I like it.

Respondent: Um, (singing continues)

**Interviewer:** Okay so can you remember the song?

Respondent: Number 1, (student sings).

**Interviewer**: Now what does, that song, what does it teach you? What did you learn from that song?

Respondents: That we should be brushing our teeth regularly.

**Interviewer**: Okay, what are those things that you don't like in that video? What are those things? You are number what?

Respondent: Number 8, how they were showing the spoilt teeth.

**Interviewer:** All the damaged teeth?

Respondent: Yes.

**Interviewer**: Why don't you like it?

Respondent: It is irritating.

**Interviewer:** Okay it is irritating to you. So what other things don't you like?

**Interviewer:** Um, how can we make the video better? What do you think we should add to the video to make it better?

Respondent: Number 1, okay, I think you should make it human, not cartoon.
So it will be like, well, visible and explanatory.

Respondent: Where they showed the picture of someone smoking. They should show, like, a picture of how they are smoking with the teeth damaged.

**Interviewer:** How can we make it better? What do you think that we should remove from the video? Somebody has mentioned where they show decayed teeth.

Respondent: I don't think that you should remove the pictures, because if they show it to others, they will understand that it is our attitude, if they are doing that, it is not good. So, I don't think that they should remove it.

**Interviewer:** Any other opinion about that.

Respondents: (*Silence).*
Respondent: Number 1, I would have preferred also if they showed practices of how they were preparing somebody’s teeth (for treatment). It would have been more understandable and like if you see the video of how they are doing it, you will feel like taking care of your teeth very well because you won’t want to be in such circumstance or situation.

**Interviewer:** Thank you. Now, would you have preferred it to be in form of drama, or do you like it as it is in form of cartoon? Or do you want it to be a form of play in that manner?

Respondents: No, yes, no, yes.

**Interviewer:** Okay. If you say, no. Why did you say that?

Respondent: Because in the picture they showed us very well.

**Interviewer**: Okay you prefer it to be in this form. Okay, any other opinion? Don't you like it to be in drama form?

Respondent: Yes, because, um, drama does not suit that kind of, um, lecture in that show.
**Interviewer:** Any other person?

Respondent: People prefer cartoons to drama; they will enjoy it the more if they leave it the way it is.

**Interviewer**: Now, we want all of you to have access to this video. How do you think we can go about it? We want you to have access to this video. So how do you think we can go about it?

Respondent: My opinion is that it should be larger or something where everyone can watch it from.

**Interviewer:** Okay, okay. Okay, I will still come back to that. You are taking me to another question.

Respondent: You can post it on YouTube, Facebook, Instagram, social media.

**Interviewer:** Okay. Social media.

Respondent: So, everyone will have assessed to download it.

**Interviewer**: Okay. What about if we want to use it for training or want to use it to train your teachers? How do you think we can use this video to train you or train your teachers? Should we be bringing your teachers to you as you are seated, so that your teachers will watch it, or show it to the whole school?

Respondent: If we watch the video, some will understand it and some may not. I prefer it that the dentist should come to the school to come and explain to the teachers, show them the pictures also.

**Interviewer:** Okay somebody is raising up his hand.

Respondent: Like, if you create a platform where students can have access and teachers, and anybody can have access to; so that you can drop the video and teach people about it.

**Interviewer:** Okay, um, will you have loved that we project the video, like projection?

Respondent: I would have loved if it projected because everyone will be able to see, to be wide enough for everyone to see, and a large crowd can see. There are some disadvantages as well.

**Interviewer:** You want to say something, you are number 15. For the projection, would you have loved it to be in your class or on the assembly, or in the school hall, where?

Respondents: Yes……mumbles

**Interviewer:** You would have preferred it in the school hall?

Respondent: Yes

**Interviewer:** Will the school hall accommodate everybody?

Respondents: Yes, no, yes.

Respondent: No, we have the junior and the senior school.

Respondent: For the senior school, it will contain us; some will just stay outside but it will contain us. It is spacious enough and it does not have place like you have to enter before you sit.
**Interviewer:** Somebody said, we can use the school hall so, but I noticed somebody said that we cannot use the school hall. Please explain.

Respondent: Population – it cannot accommodate all of us.

**Interviewer:** Okay where do you think we should use? The assembly ground.

Respondent: Maybe Yes. But the junior school will not join.

**Interviewer:** That means we have to divide you into groups.

Respondent: Yes, you show for the juniors and the seniors separately.

**Interviewer**: All right, um, and the next question says that do you like the length of the video, or you want it to be longer or shorter?

Respondent: Longer.

**Interviewer:** You want it to be longer? Why?

Respondent: Because they can explain more about it.

**Interviewer:** Okay, would you have loved it to be longer?

Respondent: I love it to be longer because, there will be more explanations. Maybe because of time they shortened it. I am not tired, and I like it and I am not fed up or felt like fast forwarding it.

Respondent: Would love it to be longer, to explain everything about how to take care of the teeth.

**Interviewer:** Okay, who has a contrary opinion? Somebody said he would prefer it longer than 13 minutes. What will you have?

Respondent: Like, um, that time is okay because some people if it is too long, they will not have interest anymore you know, they would think that it is boring and they will be less interested.
**Interviewer:** You are number what?

Respondent: Number 12

**Interviewer:** So, what number has not spoken? Number seven, you have not spoken and number nine, okay, is the picture clear enough? Do you like the graphics? Is it clear enough? Or what can you say about the graphics, the video, how it looks? What can you say about it? Is it okay?

Respondent: No, it is not okay.

**Interviewer:** Okay. Which is, is it everything, or which side is not okay?

Respondent: um… the cartoon is not okay, not big.

**Interviewer:** The cartoon is not big enough?

Respondent: Yes.

**Interviewer**: Okay. Do you like the graphics? Do you like the brightness of the video?

Respondent: Yes, number one, I would have loved it to be brighter because we couldn't see, we couldn't see the person that was, that was being…. someone was telling someone how to take care of the teeth and where he should go, we couldn’t see it was a little bit blurred.

**Interviewer**: So what about others? Do you like the graphics? Do you like the brightness or, or you don't like it?

Respondent: I don't like it because it's not, it's not bright.

**Interviewer:** Not bright enough

Respondent: Yes, because of the mouth that is.

**Interviewer:** Oh, so which one do you… want to say something? Do you like the graphic?

Respondent: Yes, but I would prefer human being to the cartoon.

**Interviewer:** But the pictures, do you like it too, the pictures of the teeth, the picture of the dental floss, the picture of the sweet, and is it explanatory?

Respondents: Yes

Respondent: I love the way they used the picture because it explains better to us and because of the picture of the damaged tooth makes one scared like ah I wouldn't want this to happen to me.

**Interviewer:** Now, you know before we watched the video, I asked you about the language. You mentioned that you like pidgin as it is the norm in our community. Now, do you prefer the pidgin, or would you want it to be in another language?

Respondents: No, we prefer the pidgin language. (*Chorused)*

**Interviewer**: Thank you. Do you now love the video?

Respondents: Yes.

**Interviewer**: Any other opinion about the video before we end the discussion?

Respondent: *Silence.*

**Interviewer:** Thank you so much. I really appreciate your participation in the discussion.
Thank you so much.

**SCHOOL THREE**

**PREAMBLE:** Introduction of the team and the students, explanation of the purpose of the meeting, giving of numbers to the students and obtaining permission to record the discussion.

**Interviewer:** Now, what are your views about the video you just watched?
What are your views? What do you think about the video?

Respondent: Number 4, I think it teaches us about how to prevent and the causes of decay.

**Interviewer:** Okay, so what are your views?
Respondent: Number 2, also how to take care of our teeth, to brush it very well.

Respondent: I am Number 8, I learnt how to brush for like, 13, 30 minutes.

**Interviewer:** 30 minutes or 3 minutes?

Respondent: 3 minutes.
Respondent: Number 5, I learnt not to eat sweet things again and we should brush after eating.
Respondent: We should not suck our fingers or tongue because our teeth will scatter.

**Interviewer:** What did you learn?

Respondent: I learnt… number 9, I learnt the accurate time to brush for 3 minutes.

**Interviewer:** Now that video, do you think is appropriate for your age group?

Respondents: Yes.

**Interviewer**: It is appropriate for your age group. Why do you think it is appropriate for your age group?

Respondent: Yes, because it taught us about oral health.

Respondent: Yes, because it teaches us how to brush our teeth. It is for young and adults.

Respondent: Number 6, because it tells us more about how to take care of our teeth.

Respondent: I am number 9. It teaches us how to prevent decays and germs from attacking our teeth.

**Interviewer:** So, it is okay for your age group. Okay. Do you like the song in the video?

Respondents: Yes.

**Interviewer:** Why do you like the song?

Respondent: Because it is about how to take care of the teeth.

**Interviewer**: Is that why you like it?

Respondent: Yes.

**Interviewer**: Why do you like the song?

Respondent: It was meaningful. It is meaningful.

**Interviewer:** Can you sing this song?

Respondent: Yes, that is okay.

**Interviewer:** Let me hear you. Do you remember the song?

Respondent: ( …*students sing*…I would brush my teeth. I would brush, when I eat…..)

**Interviewer:** Okay, maybe it is because you just heard it, that was why you were unable to sing it properly. So if you listen to it, like three times you will be able to sing it.

Respondent: Yes.

**Interviewer:** Who sang the song? So I can see that you are smiling. So, so is this part of what made it interesting to you?
What other things do you think should be in that video that will make it more interesting than what you have seen?

Respondent: Everything should be translated.

**Interviewer:** Okay, I like that. What do you think?

Respondent: It should be translated to English.

Respondent: The discussions of the doctors in pidgin should be translated.

**Interviewer:** Okay, you would have loved it to be in English.

**Interviewer:** Yes. Okay. Okay. Any other opinion, what are those things that will make it interesting to you?

Respondent: it is already interesting, because of all the kids in the video, and they also arranged the video sequence well, it is also interesting by teaching us more about how to take care of our teeth.

Respondent: It is already interesting because it shows us how the teeth are damaged.

**Interviewer**: What would make it more interesting to you?

Respondent: They can dance to the music.

**Interviewer**: You want them to dance to the music.

Respondent: Yes.

**Interviewer:** What are those things that you don't like in that video? Are there somethings that you feel should not be in the video or that you do not like in the video?

Respondent: Like that cigarette.

**Interviewer:** What about the cigarette?

Respondent: ……mumbles…..

**Interviewer:** The cigarette is just there to show you that it is not good to smoke.
It is not that we encouraged it.

**Interviewer:** Which aspects of the video don't you like?

Respondent: The damage of the teeth.

Respondent: The damaged teeth.

Respondent: The part that shows how to brush our teeth; it should have been a (human) person’s teeth.

**Interviewer:** So, what is your opinion about the damaged teeth?

Respondent: How they are showing decayed teeth is irritating.

**Interviewer:** So, you don't like it, but they need to show, to demonstrate to you that this is the good and this is bad, but you think it is not good, you don't like it?

Respondent: Yes.

**Interviewer:** Okay.

**Interviewer:** Now, which, what are the parts? What are those areas in the video that you like?

Respondent: The song.

**Interviewer:** You like the song. Okay.

Respondent: How they brushed the teeth.

**Interviewer**: That's where you like.

Respondent: The two friends.

**Interviewer:** Okay you like the two friends. Why? Why do you like it?

Respondent: How they reacted, how they interacted.
**Interviewer:** So, what do you like in the video?

Respondent: The part that showed us how to brush accurately.

Respondent: I like how the other friend is advising the other one.

Respondent: I like how the dentist talked about what can lead to teeth damage.

Respondent: I like how the other friend told his friend not to damage his teeth.

Respondent: I love the music.

**Interviewer:** Okay. Now, how do you think we can make the video better?

Respondent: It is supposed to be a lot of people in the video.

**Interviewer**: Why should there be a lot of people?

Respondent: A lot of people should be in the video so that they will all learn from the information the friends gave, they will all hear.

**Interviewer:** All right, so what should be in that video to make it better?

Respondent: They are supposed to tell the kind of toothpaste that is better for young and adult.

**Interviewer:** Now, how do you think we can distribute the video to you and your colleagues?

Respondent: They should share it through Facebook, Instagram, and platform

**Interviewer:** Which platform?

Respondent: Through social media, through YouTube, TikTok

**Interviewer:** Okay, but, but what about you? You are mentioning platform. Do you have access to phone?

Respondent: Yes, sometimes, sometimes.

Respondent: Not really.

**Interviewer:** So how can we do that because you don’t have access to phones.

Respondent: You can show it during co-curricular activities on Thursdays.

**Interviewer:** If we are to show you the videos in this school, how do you think we can do that?

Respondent: Maybe…. Maybe……. (*all the students were talking at the same time*)

**Interviewer:** One by one please.

**Respondent:** Number 8, they can show it to everybody by using projector.

**Interviewer**: Okay, okay. Somebody mentioned projector.

Respondent: You can show it to the last minute.

**Interviewer:** Okay, in that case, what are the problems that we would likely have if we wanted to show you the video using projector?

Respondent: Noise.

**Interviewer:** Noise. Who would be making the noise?

Respondents: The students.

Respondent: The lighting might affect the quality of the video being shown.

Respondent: They might not listen.

**Interviewer:** How do we solve these problems? We have highlighted about three or four problems. So how do we solve the problem of lights?

Respondent: I will say in my opinion on this we should all be gathered in a dark room.

Respondent: By controlling the students.

**Interviewer:** Okay, that will solve the problem of noise. Okay, so which other barrier did we mentioned? Participation, they might not listen. So how do you think we should take care of that?

Respondent: Inform the principal to coordinate the students.

**Interviewer:** Now, that video, will you prefer it to be in cartoon as it is?
Or you prefer it to be a form of drama? Or which method would you have wanted?

Respondent: It should be in form of a drama.

**Interviewer:** Drama? Why would you prefer it to be in the form of a drama?

Respondent: It is because it will make us understand more about it and make them demonstrate, oh, more than the cartoon.
**Interviewer:** So, do you like it as it is in the form of cartoon?

Respondents: Not really.

**Interviewer**: So, which form would you have loved it to be?

Respondent: I will prefer it to be in the normal form, with human beings.

**Interviewer:** Why is human being better than cartoon as they did for your age group?
Respondent: No, I know. It looks human.
**Interviewer:** Now will you prefer the video to be longer or shorter?

Respondent: Longer

**Interviewer:** Why would you prefer it to be longer?

Respondent: Because it will show us more things that we did not understand.

**Interviewer:** Any other person? Do you prefer it to be longer?

Respondent: Yes, because it will give us more information about how to take care of our teeth.

**Interviewer**: Okay. Now the pictures and the graphics and everything that is there, do you think it is okay?

Respondent: Yes.

**Interviewer:** What can you say about the pictures? Are they clear enough?

Respondent: Okay, it shows us how to take cake care of our oral health.

Respondent: The pictures make it more interesting.

**Interviewer**: Any other opinions about the video before we end the discussion?

Respondent: *Silence.*

**Interviewer**: Okay, thank you so much for the discussion.

**SCHOOL FOUR**

**PREAMBLE:** Introduction of the team and the students, explanation of the purpose of the meeting, giving of numbers to the students and obtaining permission to record the discussion.

**Interviewer:** What are your views about the video that you just watched? What are your views? What do you think about the video? Please state your number when talking?

Respondent: I am number 3, If you have ehm…if something wrong with your teeth go to the hospital. Don’t use battery water.

**Interviewer:** What did you learn? What is your number?
Respondent: I learnt how to take care of our teeth; we should not use battery water to wash our mouth.

Respondent: I am number 7, I learnt that we should not use our teeth to open the cover of bottles and we should not eat chewing gum, sweet.

Respondent: The thing that the video is advising us is the science of how things can damage our teeth, example smoking and chewing gum that can cause bleeding and cancer and the things that can damage our body. It also advises us to use seatbelt when we are travelling to avoid accident, which could lead to teeth shaking.

Respondent: I am number 8, I learnt that it is good to wash our teeth and not take chewing gum, not opening soft drinks with our teeth.

Respondent: I am number 1, it teaches us that we should keep out teeth neat daily.
**Interviewer:** Now, the language that was used in the video. Which language was used?

Respondent: English.

Respondent: Pidgin.

**Interviewer**: Yes, they used pidgin. Do you like that language?

Respondents: Yes.

**Interviewer:** Why do you like the pidgin? You know, I asked you the other time that which language would you prefer and you said, English. Now, the video was made in Pidgin, and you said you like it, why do you like it?

Respondent: Pidgin is like English. It is close to English.

**Interviewer:** You are number what?

Respondent: Number 3.

Respondent: Pidgin. I am number 1.
**Interviewer:** What if we make it another language, would you have prefer that? Or you like it as it is in pidgin?

Respondent: We like it as pidgin.
respondent: I am number 4, I prefer it as pidgin.

**Interviewer:** Now, do you think that video is okay for your age?

Respondents: Yes.

**Interviewer:** Is it okay/appropriate for your age?

Respondent: It is okay for our age.

**Interviewer:** Do you now like the video?

Respondent: Yes.

**Interviewer:** Okay, now what about the content of the video, all what they said and taught you in the video, do you like them?

Respondents: Yes.
**Interviewer:** Why do you like the contents?

Respondent: Because the person that taught us used pidgin language that we understand and that is why I like it.

**Interviewer:** So why do you like the contents?

Respondent: Number 5, because the things I did not know before I learnt it now.

Respondent: I like it because it is educative, and it gives us the awareness about what we don't know before about our teeth.

Respondent: I am number 3, how they are speaking and showing us the picture.

Respondent: I like the video because it teaches us how to maintain our teeth, things that can lead to damage of our teeth and they showed us dirty mouth and the things that are bad in our mouth.

**Interviewer:** Now do you… … Do you like the song?

Respondents: Yes, yes.

**Interviewer:** Why do you like the song?

Respondent: I like it because it is fun.

Respondent: It is, it is very interesting.
Respondent: It is very neat. It is very new.

**Interviewer:** Can you sing the song?

Respondents: *Students sing……*

**Interviewer:** So, you are interested, you are interested in the song?

Respondents: Yes.

**Interviewer:** Do you remember it?

Respondent: Yes.
**Interviewer:** Okay. Now, what are those things that you don't like in that video? Are there some things that you don't like that you want us to remove from the video?

Respondent: The smoking……….

**Interviewer:** They just used that picture that you should not smoke, it is not that we encourage smoking. So, what are those things you don’t like?

Respondent: We like, we like everything.

**Interviewer:** That is your view.

Respondent: I don’t like how they are showing the chewing gum. …. (*Laughs).*

**Interviewer:** That is her view. You don’t like how they are showing chewing gum and sweets.

Respondent: Everything.

**Interviewer:** You like everything.

Respondents: Yes.

**Interviewer:** Okay. Now, what about those things that you think we should add to that video.

Respondent: Maybe how they performed, how to clean the disease of the teeth. How to make use of that thing that looks like spanner.

**Interviewer:** Do you mean dental floss?

Respondent: Yes, used to remove all dirt from our mouth.

**Interviewer:** They should demonstrate it.

Respondent: Yes.

**Interviewer:** Any other thing you think we should add to the video?

Respondents: *Silence.*

**Interviewer:** Okay, this video, how do you think we can distribute it to you students, your teachers, and your parents?

Respondent: I am number 10, you can post it on Facebook, and you can send it to our principal and he would send it to the teachers and our parents.

Respondent: I am number 3, we can give you our WhatsApp number.

**Interviewer:** Do you, students, have WhatsApp numbers?

Respondents: Me, I have a WhatsApp number.

**Interviewer:** So how do you think we can send it to your parents?

Respondent: Maybe we should give you their numbers.

Respondent: You can create a group or to tell them to search it online.

Respondent: Some parents do not have phones, maybe you give us notes that will advise us and public enlightenment.

Respondent: Yes…*laughs*…because some parents and students do not have phones, we can distribute it to our teachers as a form of channel.
**Interviewer:** Thank you. Now, will you prefer cartoon or drama, or which other method will you prefer or do you like it as it is?

Respondent: Number 5, I prefer cartoon because most of the children watch cartoons on YouTube or Netflix.

Respondent: No 3, I prefer cartoon.

**Interviewer:** Will you prefer drama to cartoon?

Respondent: Yes, No.

Respondent: I prefer drama because it will appear more real to people. It will be more like reality to people than cartoon.

Respondent: I prefer drama because it will make us to gain something but in cartoon, we may not hear some words and drama will advise us how we can participate in that video.
**Interviewer:** Now if we want to show the video to all the students in the school, how do you think we can do it? Your number?

Respondent: By giving it to the principal and he will give it to the teachers and to our parents.

**Interviewer**: This video that you just watched; how do you think we can show it to all the students?

Respondents: By projecting, by projecting.

**Interviewer:** By projecting it. Do you understand what she meant by projection? Why would you want projection? Why?

Respondent: Number 3, because projection is very large, so everybody sees it, everybody sees.

Respondent: I prefer projection because all the things that is showing in that video will be shown in the projection.
**Interviewer:** Would you have preferred the video to be longer or it should be shorter, or is it okay, as it is?

Respondent: It is better like that.

Respondent: No.

**Interviewer:** Why?

Respondent: Because it will take much of the time if it is longer.
**Interviewer:** Is the video interesting to you?

Respondent: Yes.

**Interviewer**: Why is it interesting?

Respondent: I liked the video because of the music and the character.

Respondent: Because of the way they showed us how to brush our teeth.

Respondent: Because of the information that was given to us.

Interviewer: Now, what about the pictures? Are the pictures, appropriate?

Respondents: Yes.

Respondent: I am number 8, Yes.

Respondent: I am number 7, it is okay.

**Interviewer**: So, what are the other interesting things that you think we should add to that video?

Respondent: I also like the music, add more and let the music be longer.

**Interviewer**: Okay, thank you so much for participating in the focus group discussion. Are you elated? Are you happy?

Respondents: Yes.

**Interviewer:** Do you like the video?

Respondents: Yes.

**Interviewer**: Any other opinion about the video before we end the discussion?

Respondents: *Silence.*

**Interviewer:** Thank you so much for your participation

**SCHOOL FIVE**

**PREAMBLE:** Introduction of the team and the students, explanation of the purpose of the meeting, giving of numbers to the students and obtaining permission to record the discussion.

**Interviewer:** What are your views about the video that you just watched?
I believe you still remember your number?

Respondent: Number 2. In my view everything is exactly what am doing right or wrong, and I think I have learnt a lot.
**Interviewer**: What did you learn from the video?

Respondent: I learnt so much in my life.

**Interviewer**: Like?

Respondent: Not using my teeth to open bottles, not using toothpick and not chewing gum.

**Interviewer:** What did you learn from the video?

Respondent: I learnt a lot, like not to be smoking, um, not to be chewing sweets or chewing gum, not using toothpick or sharp object in our mouth. I learnt a lot even the things I don’t expect when taking care of the mouth.

**Interviewer:** So, what did you learn?

Respondent: I am number 7, I learnt that we should brush our teeth and not to use our teeth to open cover of minerals *(soft drinks)* and we should eat fruits and vegetables.

**Interviewer:** So, what do you learn, any other person?

Respondent: *Silence*

**Interviewer**: Thank you. Do you think the video is okay/appropriate for your age group, is it appropriate for your age group? Yes, or No?

Respondent: Yes, it is appropriate for our age group and for the old ones. The reason is that our old ones, some, cannot read well; through the video, even if they cannot read, seeing the video they will learn that these are what they said we should not do and do, and it will help them a lot.

Respondent: I am number 5. This video is very appropriate for us in this our stage because there are so many things that our mates are doing that may be corrected. It is also appropriate for the others.

**Interviewer:** Thank you. Any other opinion, is it okay for your age group?

Respondent: Yes. (*Chorused*)

**Interviewer:** Why? Why do you think it appropriate or okay for your age group?

Respondent: Because some of our age groups are smoking, they eat some bad sort of things.

**Interviewer:** Why do you think it appropriate or okay for your age group?

Respondent: *Silence*

**Interviewer:** Okay, what are those things that you don't like in that video?
Maybe there are some things that you don't like in that video. Is there anything you don't like?

Respondent: No, there is nothing we don't like.

**Interviewer:** That means you like everything that was shown in the video. What about the graphics, the pictures, or those that are…the contents? Do you like it? Or are there some that you don't like in that video?

Respondent: There are some I don't like especially when they looked inside the mouth of the decayed teeth.

Respondent: Like, they do it like cartoon.

**Interviewer:** Which one do you like if they don't use it as cartoon?

Respondent: Human beings.

**Interviewer**: Okay. Human beings. Why do you think human being is better than cartoon? Does any other person think that human being is better than using cartoon?

Respondent: *Silence***Interviewer:** Or you like it as it is?

Respondent: Yes.

**Interviewer**: Do you like the song?

Respondents: Yes.

**Interviewer**: You like the song? Why? Why do you like the song?

Respondent: It is. It is so, fun, like, it is okay.

**Interviewer:** Can you sing the song? Do you, can you remember the song?
Can you remember?

Respondents: *(Students sings….)*

I go brush. I go brush very well.

I go brush. When I show to this. I go brush so much.
I go brush. I go brush. I go brush. I go brush well well.

**Interviewer**: That means you like the song because you can still remember it?
Respondent: Yes.

**Interviewer:** How would you like us to show you that video? Is it in the class or in your school hall or on the assembly ground? Which way do you think we can show you so that others too can benefits from it?

Respondent: In the school, in the school hall

**Interviewer:** Do you have hall that can contain everybody?

Respondents: Yes.

**Interviewer:** Do you prefer assembly, which venue?

Respondent: I am number 2, I prefer assembly because um… everybody will be on the assembly and will be able to watch.

**Interviewer:** How many minutes is the video? Did anybody take note of it?

Respondent: No.

**Interviewer**: It was not up to 30 minutes. So will you prefer it to be longer or shorter?

Respondent: Longer sir.

**Interviewer:** So why do you think it should be longer?

Respondent: Because of the benefit of more information to us.

Respondent: I think it's okay like this, it is not too long or too short and we achieve lessons from it.

**Interviewer:** So how do you think we can share this video to you students and your teachers?

Respondent: Through media like WhatsApp, YouTube etc

**Interviewer:** You are number what?

Respondent: Number 9.

**Interviewer:** Any other opinion on how we can share the video to you?

Respondent: I am number one, you can share the video by posting online like Facebook and so on.
**Interviewer:** Any other opinion on how we can share the video to you?
Respondent: *Silence*

**Interviewer**: Any other opinions about the video before we end the discussion?

Respondent: *Silence*

**Interviewer:** We have come to the end of our discussions. Thank you so much for your time.

**SCHOOL SIX**

**PREAMBLE:** Introduction of the team and the students, explanation of the purpose of the meeting, giving of numbers to the students, and obtaining permission to record the discussion.

**Interviewer:** Good morning, everyone again. How are you today?

Respondents: We are fine.

**Interviewer:** As we have introduced ourselves earlier, that we are dentists from the University College Hospital, we just wanted to know your views about the video that you have watched.

**Interviewer:** From the video you watched what did you learn? What does it mean to you?

Respondent: My name is…

**Interviewer:** No! Please do not mention your name, just tell me your number.

Respondent: I watched the video, and it teaches me about how to take care of myself, how to take care of my teeth and how to prevent our body and how to prevent my teeth from teeth decay, teeth damage, teeth scatter and then the video taught me how to, how to meet the dentist and seek for advice and that I should not use self-medication.

**Interviewer:** Okay, you said how to prevent tooth decay, what about prevention of tooth decay? What are the things that you are supposed to do to prevent tooth decay?

Respondent: By not eating sugary things, by washing your teeth twice daily and by visiting the dentist at least 6 months, every 6 months.

**Interviewer**: Is there anybody else who wants to say something?

**Interviewer:** What about the effect of other things? Can you tell us from what you have learnt how will you prevent scattered teeth?

Respondent: How to prevent scattered teeth are by not opening bottle with my mouth, by not using my teeth to cut my fingers nails, by not using my tongue to scratch my teeth.

**Interviewer:** Thank you very much, any other person?

Respondent: It teaches how to prevent tooth decay, mouth odor.

**Interviewer:** And you will tell us how to prevent the mouth odor, tooth decay.

Respondent: It will prevent us from mouth odour, by not using knife to do our teeth to prevent mouth scatter. By using our hand to lick our tongue, it prevents mouth scatter and the licking of sweet to prevent mouth decay.

Respondent: Number 3, the video teaches me how to take care of my teeth and teaches me about gum disease.

**Interviewer:** How do you clean your teeth?

Respondent: Twice in a day with a brush and the toothpaste, which contains fluoride.

**Interviewer**: And what type of toothbrush?

Respondent: Medium toothbrush, and the dental floss to take out dirt from my mouth and not eating a lot of sugar and taking care of my teeth.

Respondent: From the video I watched, I learnt how to take care of our teeth to prevent gum disease and I learnt that we should not smoke, and I learnt about tooth decay, by using the toothpaste I learnt how to take care of tooth decay, by brushing our teeth three times, by visiting the dentist that's what I learnt.

Respondent: I am number 5, how to prevent us from teeth scatter, not to play rough in the school.

Respondent: It prevents our teeth from teeth scattering and we should not play rough in the school, not to fight each other, for tooth decay, stop eating something like biscuits, sweet, cake, that it contains sugar, it affects our gum.

Respondent: I am number 6, I learnt that tooth decay is caused by many things, not eating too much sugar, that we should brush our teeth twice daily, we should use toothbrush, a medium toothbrush, toothpaste that contains fluoride, we should not be playing rough in the school.

**Interviewer:** Why should you not be too playful?

Respondent: Somebody can just push you and you could just fall.

**Interviewer:** So, what about it? What if you fall? What can it cause when you fall?

Respondent: It can cause damage to our teeth.

Respondent: The video teaches about how to prevent our teeth from decay, we should not be chewing gum, we should not be using our teeth to open bottles and we should not be playing rough in the school.

**Interviewer:** Why should you not play rough in the school?

Respondent: Because it can cause scattered teeth and the teeth can fall off.

Respondent: Number 8, what I learnt from the video is about how to take care of my teeth.

**Interviewer**: How do we take care of our teeth?

Respondent: By washing our teeth twice daily, not using blade to remove dirt in our mouth, and not using any sharp objects

**Interviewer:** To do what?

Respondent: To remove the dirt from our teeth and using …umm.

**Interviewer:** What should we use instead?

Respondent: dental floss.

Respondent: And my fingers nails; that I should not be using my teeth to cut my nails and I know using my teeth to open bottle of minerals, by not eating sugary, by not playing rough.

**Interviewer:** Okay, any other thing?

**Interviewer:** The next question is when we are talking about the video, is that video appropriate for the students of your age? Why do you think it is appropriate?

Respondent: Number 1. The reason why I think it is appropriate is that the video teaches a lot, which is the first lesson I learnt from the video, is that by not using self-medication because the guy the guy that has the teeth problem in the video says someone gave him ehmm…a homemade battery water so he has used that battery water maybe he has used self-medication and I also heard he asked his friend to bring the drug his parents used maybe it is going to cure his own problem or so. The video teaches me not to use self-medication and if I have problem I should move to my doctor and my dentist

**Interviewer:** Did you understand the language of the video?

Respondent: Yes, I do.

**Interviewer**: Which of them, which of them did you watch? Which one do you think we should do better?

Responding: The first one they played, the Pidgin I understand of course, I understand and I was not not there when they played the second one in Yoruba.

**Interviewer:** So, was the one you watched in Pidgin English?

Respondent: Yes.

**Interviewer:** So, what do you like most about the video?

Respondent: What is good about the video is that the moral lesson the video taught us is what I like.

Respondent: I like the language, it is okay.

**Interviewer:** What about the cartoon? Is that cartoon okay for your age or would you have preferred human characters? Or would have preferred the whole thing should be cartoon or should be younger people?

Respondent: Everything is okay.

**Interviewer:** You like it the way it is.

Respondent: Yes.

**Interviewer:** What about the song at the end, does it make sense?

Respondent: Yes.

**Interviewer:** Would you have preferred the song this is the way I brush my teeth….?

Respondent: Everything is fine.

**Interviewer:** Why is everything fine?

**Interviewer:** What about the song? What do you know about the song? Can you sing the song? Why is everything fine? Can you remember the song?

Respondent: Everything is fine.

**Interviewer**: *….smiles*

**Interviewer:** We have to know what is fine and what is not fine.

Respondents: ….*murmuring*

**Interviewer:** Number 2, okay from there you can speak. So what did you like from that video? How it was projected? Would you have preferred that human beings act the video instead of us playing it on screen? Would you have preferred it as drama, or did you like how it was done as a cartoon instead of human characters? Just tell us what you like about the video.

Respondent: I like the way they did the video.

**Interviewer:** How did they do in the video that you like?

Respondent: How you used the cartoon.

**Interviewer:** How they used the cartoon?

Respondents: ……. *Murmuring*

Respondents: We love it.

**Interviewer:** What about the content?

Respondent: We like it.

**Interviewer:** What else do you want us to include in the video?

**Interviewer:** Do you understand it well?

Respondent: Yes.

**Interviewer:** Was it difficult?

Respondent: No.

Respondent: I think the video was not well mentioned.

Respondent: I think the video is not very important because it would have been good if it was human performance. Firstly, not all students understand the language but the content was okay and it passed the message but it would have been good if it was human performers.

**Interviewer:** Human beings that acted like a drama or we should change the cartoon and put human characters, or you want us to act it like a play-drama?

Responding: No, change it to human form…………...

**Interviewer:** The song. What about the song? Do you like the song?

Respondent: The song is good.

**Interviewer:** Do you know the song?

Respondent: I don't remember

**Interviewer:** That means you don't like this song.

Respondent: I like it.

**Interviewer:** So, tell us what is good about the video.

Respondent: The content was good

**Interviewer:** Okay.

Respondent: And how it was passed across.

**Interviewer:** So, what did you not like about the video?

Respondent: First, need to change the language, how the cartoons are talking.

Respondent: I like how they performed, and I don't think it can be human to human because some people will be making noise when it is human to human. When it is on TV that everybody is watching, so it can be it can be interesting, and I can see that is well performed.

**Interviewer:** What else do you like about it?

Respondent: So, I like the content and how the pidgin they are speaking.

**Interviewer:** Do you prefer the Pidgin to Yoruba?

Respondent: Yes, I like how it teaches about how to take care of our teeth from tooth decay and gum disease.

**Interviewer:** What areas do you not like in the video?

Respondent: There is nothing bad about the video.

Respondents: I like how they performed the drama, I like how they performed the drama and because the content was good. I like how they performed the drama. Secondly the contents are good, they told us not to....
**Interviewer:** What don't you like about the video?

Respondents: Nothing is bad about the video.

Respondent: Nothing is bad about it. They are good contents and the drama is also interesting and that is it.

**Interviewer:** So, is the language appropriate? Or do you prefer English to Yoruba.

Respondent: English is better. English is better, English and pidgin are good.

**Interviewer:** You prefer Yoruba?

Respondent: No.

**Interviewer:** Any other person?

Respondent: I like all the things in the video.

**Interviewer:** What have they said or what have they not said that you want them to say? What is good about it and what is not good about it?

Respondent: Everything was good.

**Interviewer:** Or what will you want us to add to it?

Respondent: Everything is good, the performance, everything was good.

**Interviewer:** What will you have us to add to it if we are to bring it to any other time?

Respondent: *Silence*

**Interviewer:** So, does anybody have anything to add?

Respondent: I like the video because there are two persons; one is asking and one is answering, so like other people they will just want the one person, you just be doing everything but those two persons in the video are okay.

Respondent: Like some of us do not understand the language so we prefer it the local language.

Respondent: Noting is bad about it.

**Interviewer:** What is good about it in what you have mentioned or is there anything that you want us to add to it?

Respondent: I like the video because I like the way they interacted; the doctor asked the question and the dentist answers immediately and orderly, that's what I like about the video.

**Interviewer:** What don’t you like about it?

Respondent: The color and ehmm…. Because.

Respondent: For me because I just don't like the color.

**Interviewer:** What don't you like about it?

Respondent: Because it is somehow dull.

**Interviewer:** Is it something that is so sharp?

Respondent: yes, yes, yes.

**Interviewer**: Is that not bright? What about the audio, the voice? Is the voice clear enough?

Respondent: Yes, it is okay. Thank you.

**Interviewer:** So, if we have to bring this to the school activities, will you prefer us to show it like this or will you prefer us to send it to your phones? Which of the two will you prefer? Or should we send it to your teachers?

Respondent: Do you want to bring it to the school?

**Interviewer:** Yes, we want to bring it to the school, formally. Do you want us to send it to you or do you want us to send it to your teachers and send it to your parents? Or do you want us to send it to your parents to send it to you or do you want us to send it to your teachers to send it to you? Or do you want us to project it the way we projected it this morning?

Respondent: You come and do it the way you did it this morning because if you say that you will send it to the teachers, not every parent has big phones.

**Interviewer:** But your teachers can show you or do you want us to come and project it?

Respondents: Yes.

**Interviewer:** You know he mentioned a lot of things in the video, did you understand some of those things better when the dentist was talking or responding to the interview or like which part?

Respondent: Ehm… how to take care of our teeth.

Interviewer: Was it the part that was showing you how to brush your teeth?

Respondent: How to brush, how to remove the dirt from our teeth from our teeth.

**Interviewer:** Which part of what the dentist said was not in the video?

Respondent: Nothing.

**Interviewer**: So, this video that you just watched, do you prefer the way it is or you would have preferred a drama was acted? You know there was a little bit of drama, one drama like people act in the normal home video.

Respondent: This one is okay

**Interviewer:** Why do you think it is okay?

Respondents: Because human beings cannot act like computer.

**Interviewer:** Why did you say human being cannot act like computer?

Respondent: The reason why I said the video is okay is that when the doctor and the dentist were discussing, they were showing us some teeth some… maybe rotten teeth. If they are giving us drama, they won't be able to show us that.

**Interviewer:** So, what else, who has something else to say about it?

So you said everything is correct and it is appropriate. So let me recap what you said, you all said the video was appropriate for students and you told us about different things that you learnt from the video, also the good things about the video according to some was that ehm…it was cartoon-like, you like the audio, the cartoon while some people said they would have wanted the two languages, some prefer it in Yoruba to the other languages. Some did not like the color, that it was too dull, some would have preferred brighter ones. What other things that you mentioned have I not captured?

Respondent: Some preferred human forms to cartoon.

**Interviewer:** Okay, some preferred human forms compared to cartoon.

**Interviewer:** So, what do you think about the length of the video, is it too short or too long? What are your thoughts, everybody?

Respondents: it is normal, it is normal.

**Interviewer:** Why did you say it is normal?

Respondent: It is normal because all what we need to understand you showed us so it's not too long.

**Interviewer:** Who think that it is too short?

Respondent: It is just appropriate.

**Interviewer:** So, if we make it longer and include some other things, will you like it?

Respondents: No, yes, no.

**Interviewer:** What things will you love us to include?

Respondent: You know the victims that has the teeth problem, maybe they should add where they are doing the operation for them.

**Interviewer:** Thank you. Any other thing you want us to include?

Respondent: *No response.*

**Interviewer**: Any other opinions about the video before we end the discussion?

Respondent: *Silence.*

Thank you so much for your responses and attention.

**TEACHERS FGD AFTER VIDEO PROJECTION**

**PREAMBLE:** Introduction of the team and the teachers, explanation of the purpose of the meeting, giving of numbers to the teachers and obtaining permission to record the discussion.

**Interviewer:** From the video you watched today, what can you say is your experience in using the video to communicate to the students?

Respondents: Please the question again?

**Interviewer:** The question is what is the best way to use the video to communicate to the students?

Respondent: I thought you made use of a projector instrument; it is a very good program. We can also use that to teach our students and also, we can make use of phones like our Android phones.

**Interviewer:** Who has anything to add to it?

Respondents: Showing the video to them will make them to be more more… they will be able to participate because they all believe in watching films; when they introduce video, this will allow them to pay attention, and they too will want to participate.

**Interviewer:** That is what I also observed because when we projected the teeth, they all shouted and they rejoiced that this is this and that but we would like you to inform us what our shortcomings are and what and where in the video that we have shown and in the program that we can always do better.

Respondents: What I can say is that using that video you can still examine their teeth privately.

**Interviewer:** Do you want to say something sir?

Respondent: *(Voice not clear)*

Respondents: I don't even have suggestions because you are professionals.

Respondent: Anyway when I was in secondary school, I am very sorry for sharing this, I had problem with my teeth at the right side at the corner of my teeth so I went to the hospital at ….so they removed it but if we had this kind of program during my secondary school days, I would have known what to do. I did not know that the sugar that we were eating could cause problem to our teeth then. So by you bringing this program now, the program will educate and orientate the student, which is very impressive.

Respondent: What you have just said now brought my awareness to a bitter experience that I had with my tooth when I was a teenager. We were playing football and it got to a time when my side won the game and I was standing on the other side of the pitch making jest of the others so one of them threw a stone at me and it hit me on my teeth. Unfortunately, I did not inform my parents and the gum started swelling up……. What I am saying in essence is that the students should be made to know that whenever they have problems with their teeth, they should report immediately as shown in the video.

Respondent: In addition, most of the students find it difficult to read but looking at the video it can help them.

**Interviewer:** Thank you, sir, the information that I picked from your words is awareness. One of the things that we should try to do is more awareness. Are there other things that you think this form of education should focus on? We have talked about hmmm… awareness. Are there other things that when we are teaching, we should mention?

Respondent: In addition to that, you made mention of something like an orientation, something happened in the morning that they learnt that removing the bottle top with their teeth; because as you are looking at me, I do it as well, but I think we can also orientate our students not to do that.

**Interviewer:** Thank you. We are looking at how this can be sustainable. The truth is that we cannot be going to all the schools to show them the videos, the question is that how can we disseminate this video working with teachers? Even if we are just sending videos to the school will it work, how can that work?

Respondent: That is very easy. We have a platform, a platform called ….. and this platform includes all the teachers in the state. So with that single platform, we can disseminate the information so all can have access to it. We can disseminate it through ….. that is association of principals of secondary schools so you can disseminate it through principals in the state.

**Interviewer:** Are there challenges as regards that because there are a lot of videos sent on WhatsApp that do not reach the target audience? How are we going to ensure that it gets to them or is there need for incentives that will facilitate it, that will make sure that they are really making use of the videos in the schools?

Respondent: In each school, you should find a representative so that if the video is sent to their platform, those representatives are the ones that will anchor the activities and they will be the one that will impart the knowledge to the students.

**Interviewer:** Somebody that will be held responsible. Are there other things, are there legal issues or curricular concerns, I don't know… that are associated with this? Are there permissions to be taken or can the principals just decide?

Respondent: In basic science, we teach the care of the body. In the care of the body, one aspect is teaching them how to take care of their mouth.

**Interviewer:** It means that this can be incorporated into that curriculum.

Respondent: I think the government can also include it in the curriculum.

**Interviewer:** Thank you. You have watched the video today and it is basically talking about care of the mouth and not care of the other parts of the body. Is there anything special about it that is different from…

Respondent: I think we already have it in the curriculum like I was taught the incisors.

Respondents: Yes, yes, yes, so it's already there.

Respondent: This practical part is the one that we cannot do.

**Interviewer:** So now if this video is sent to you, how will you let them (students) know about what you saw in this video?
Respondent: This morning I seized an android phone from a student and I discovered that the student was watching TikTok and the girl was playing the video for like 30 minutes. I was surprised. In that case, if they (students) can do that, it means this one too will work, it will work.

Respondent: Before, you said you will send the video to the teachers……..
**Interviewer:** Yes, but now we projected the video, and everyone watched it. Everyone cannot watch it from their phones. Are there facilities needed for class sessions? Are there challenges?

Respondent: There will be challenges because even the ………………….it is a great challenge.

**Interviewer:** So, it means that now that we have made the video, to access it by the students is now the issue. How do you think we can address that?

Respondent: As regards that, I think like four years ago I went to a program, a seminar and after the seminar they gave us a disc and, in that disc, when I watched it, it was full of all what we have been taught in that seminar. So, I don't know if hmmm you can provide a disc because many have DVD players at home.

**Interviewer:** So, do you mean that we should make this inform of… disc and it will get to their various homes?

Respondents: *(all confirming the statement yes yes yes yes) smiles and laughs....*

**Interviewer:** Thank you so much. We are rounding up in 10 minutes. Now, what you saw in the video, was it useful overall?

Respondents: Yes, it is very interesting and very educating, especially the Yoruba aspect because you interpreted it in Yoruba and the students were very impressed.

Respondent: That means as we are talking and talking, majority of them will not concentrate but just because of video, they will want to know what the people are saying.

**Interviewer:** So, we are also making sure from our own side that what we are teaching is appropriate for the students. In this case, would you say that the terminologies are appropriate for the students?

Respondent: What is there is the interpretation of the English.
Respondent: I don't think there is another way to come down to their level than speaking in their own mother tongue, I think that will be enough.

Respondent: Whatever terminology you use in English has been interpreted in Yoruba.

**Interviewer:** Thank you. Does anyone have something to add?

Respondent: I have learnt how we are to take care of our teeth, how we are going to remove the germs, it is from here that I learnt that treating our teeth, our mouth every three months and we should not be using all these harmful things that we are using to remove food particles from our teeth, we should be using the recommended tools.

**Interviewer:** Thank you very much. Thank you so much for your time. We really appreciate you for all what you have said about the video.
