# Supplementary figures and images for: Development and validation of School Oral Health Promotion Program “SOHEPP” oral health video for the training of adolescents and teachers in Ibadan, Nigeria
Source: PLOS Glob Public Health. 2025 Apr 22;5(4):e0004521. doi: 10.1371/journal.pgph.0004521 (PMC12013892; doi:10.1371/journal.pgph.0004521)

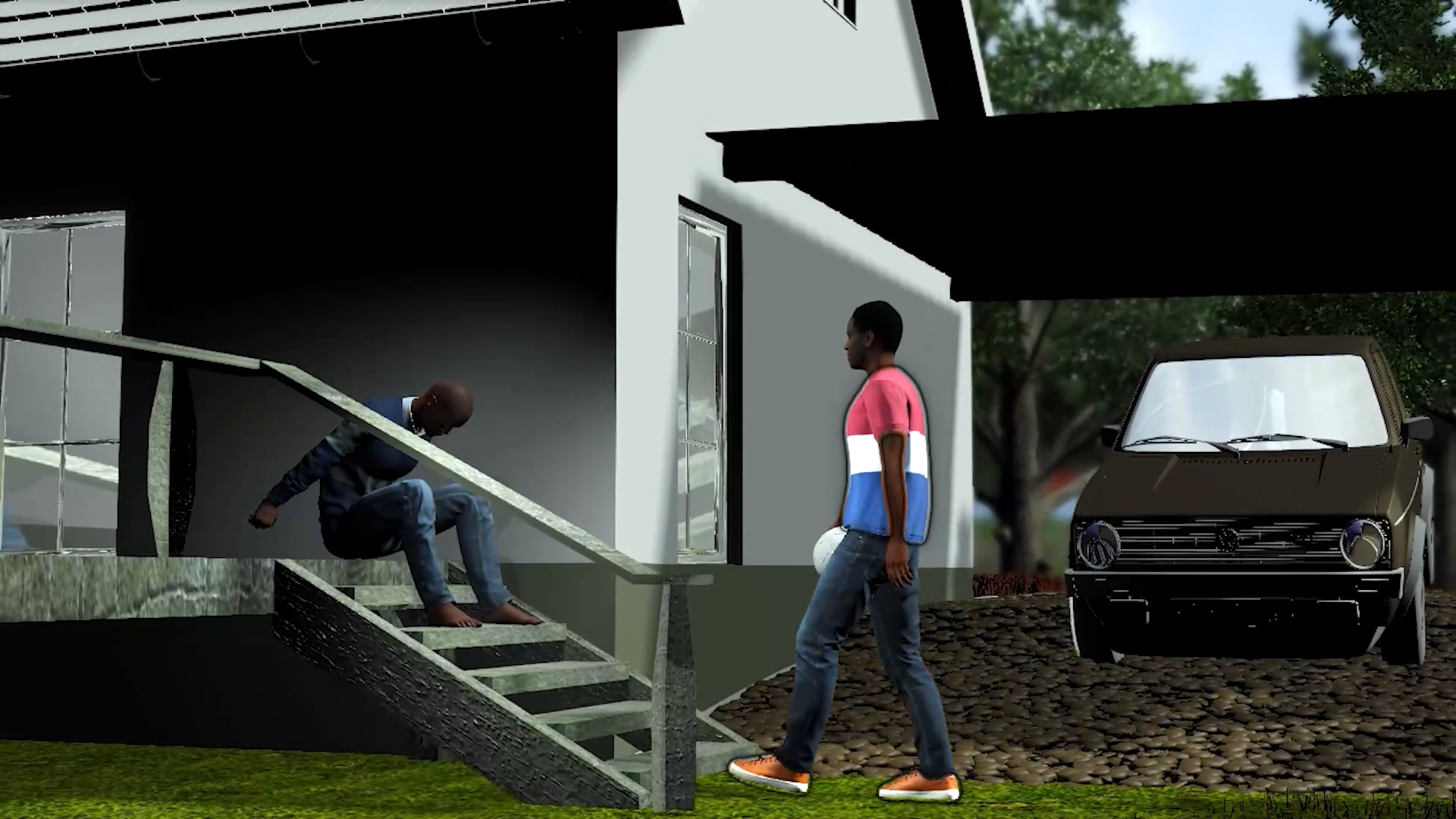


S1 Appendix: *Seun* holding a ball in his hands and walking into the compound of the house of *Ade*

Supplement: S1 Appendix — (DOCX) [file pgph.0004521.s002.docx]

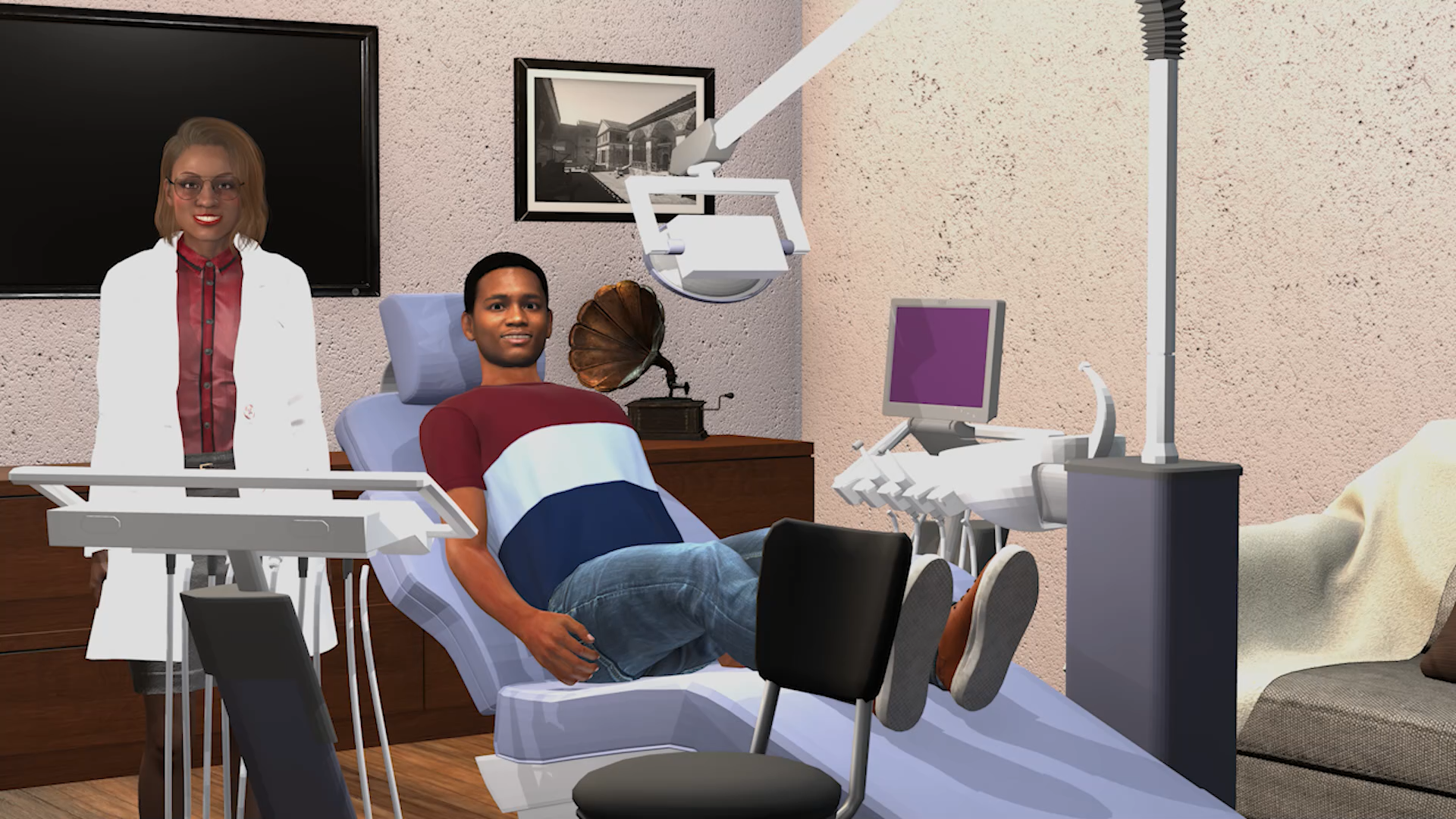


S2 Appendix: *Seun* recounting experience with the dentist

Supplement: S2 Appendix — (DOCX) [file pgph.0004521.s003.docx]

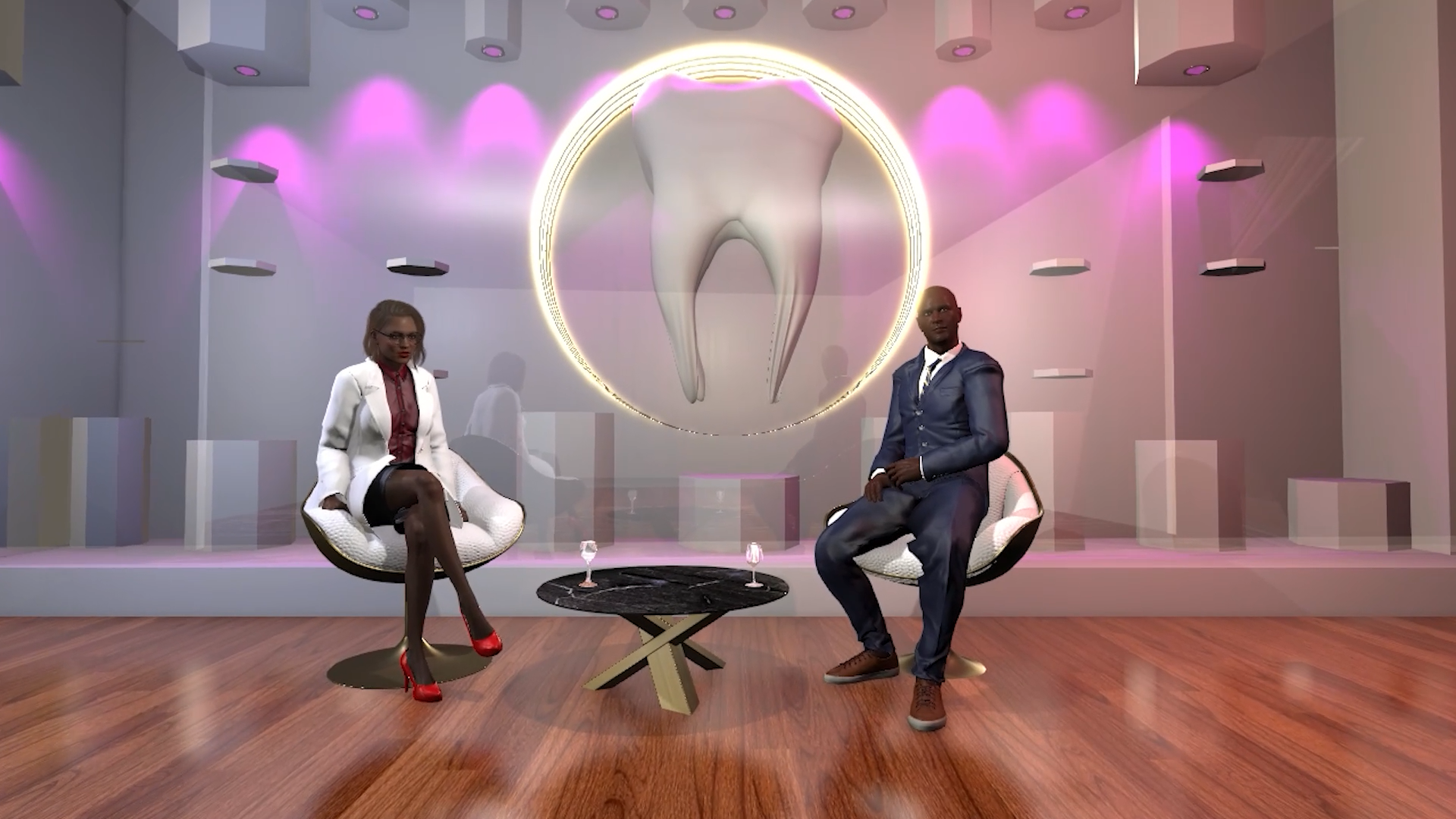


S3 Appendix: Interview of *Dr Fresh*, a female dentist

Supplement: S3 Appendix — (DOCX) [file pgph.0004521.s004.docx]
