# Supplementary material for: Development and validation of School Oral Health Promotion Program “SOHEPP” oral health video for the training of adolescents and teachers in Ibadan, Nigeria
Source: PLOS Glob Public Health. 2025 Apr 22;5(4):e0004521. doi: 10.1371/journal.pgph.0004521 (PMC12013892; doi:10.1371/journal.pgph.0004521)
Supplement: Attachment — Submitted filename: Reviewe1.pdf [file pgph.0004521.s005.pdf]

Reviewer's comments

Line 36-37: Presently, in Nigeria, like many other developing countries, oral health promotion programs remain the responsibility of the public health dentist.

Revise the sentence to: Presently, in Nigeria, as in many other developing countries, oral health promotion programs remain the responsibility of public health dentists.

This phrasing removes "the" before "public health dentists," making it clear that the responsibility is generally held by professionals in this field rather than a single individual.

Line 47-50: The video was developed by a team of experts from various specialties, which included public health dentist, pediatric dentist, epidemiologist, children's illustrator, communication scientist, animation expert, oral and maxillofacial surgery trainee, pediatric surgeon, and science educator.

If the authors are unaware of the number of individual experts who developed the video, the sentence should be revised to:

The video was developed by a team of experts from various specialties, which included public health dentists, pediatric dentists, epidemiologists, children's illustrators, communication scientists, animation experts, oral and maxillofacial surgery trainees, pediatric surgeons, and science educators.

This version avoids any ambiguity about the number of specialists involved from each field.

Line 64-66: It would be beneficial to define "adolescent" and state the age range for clarity, especially given that adolescence is central to the study. Defining this term and age range can help readers, particularly those unfamiliar with the exact age group, understand the context and focus of your study.

Adding a definition can be done briefly and naturally, such as: "Oral health conditions and practices vary as an individual journeys through the different stages of life, such as adolescence (1), typically defined as individuals aged 10 to 19 (World Health Organization). This period is characterized by pronounced social and psychological vulnerabilities, which can profoundly impact an individual's well-being and development (2)."

Including this information will make the introduction completer and more accessible for readers unfamiliar with specific definitions.

Line 150: In addition, it also taught skills on effective tooth brushing habits. The right sentence should be "In addition, it also teaches skills on effective tooth brushing habits."

Using "teaches" maintains a consistent present tense, which is commonly used when describing the content of educational materials.

Line 162-165: He described the sleepless nights he endured, the ineffectiveness of medications and the various harmful substances he had tried such as alcohol, bitter leaf, salt, and "battery water" (water used to refill lead-acid batteries), which only worsened his condition.

For improving clarity and providing context, here is a revised version of the sentence:

“He described the sleepless nights he endured, the ineffectiveness of medications, and the various harmful substances he had tried, such as alcohol, bitter leaf (Vernonia amygdalina, a vegetable widely eaten in some parts of Nigeria), salt, and car battery water (water used to refill lead-acid batteries), which only worsened his condition.”

This version provides additional context about bitter leaf, making it more accessible for readers unfamiliar with it, and clarifies "battery water" to avoid confusion.

Line 181-182: Language experts checked the script for correctness and translated it into Pidgin English and Yoruba.

Change the above sentence to: Language experts checked the script for correctness and translated it into Pidgin English (a widely spoken English-based creole in Nigeria) and Yoruba (a major indigenous language spoken in southwest Nigeria).

This version makes the sentence more informative and accessible.

Line 252: You mentioned focus group discussions without an abbreviation, and in line 267 you used the abbreviation “FGD.” Please, make a correction by writing the abbreviation FGD for focus group discussions in line 252**.**

Line 346: The conclusion should include a policy statement or policy implication. An example of such statement could be **“Given the positive reception of the oral health video by adolescents and teachers, policymakers could consider incorporating such multimedia tools into school curricula as part of a standardized oral health education program.”**

Line 373: References

In most academic styles, it is best to avoid mixing capitalization styles within references in a single manuscript. That is what you have in your manuscript. See below:

Lawal FB, Taiwo JO. Making a Case for Formal School-Based Oral Health Promotion: Oral Health Knowledge, Attitude and Practices of Pupils in Ibadan, Nigeria. *Int Q Community Health Educ.* 2018 Jan;38(2):99–105

Popoola BO, Dosumu EB, Ifesanya JU. Periodontal status and treatment need among adolescents in Ibadan, Southwestern Nigeria. *Braz J Oral Sci.* 2015;14(2):117–21

Using both uppercase and lowercase formats within the same manuscript without a clear rule or pattern may appear unprofessional. To ensure consistency, choose a reference style guide and apply it uniformly across all references in the manuscript.

Also, in reference number 15, remove the double quotation marks since other references do not have them.
