# Supplementary material for: Development and validation of School Oral Health Promotion Program “SOHEPP” oral health video for the training of adolescents and teachers in Ibadan, Nigeria
Source: PLOS Glob Public Health. 2025 Apr 22;5(4):e0004521. doi: 10.1371/journal.pgph.0004521 (PMC12013892; doi:10.1371/journal.pgph.0004521)
Supplement: Attachment — Submitted filename: Response to reviewers comments.docx [file pgph.0004521.s006.docx]

**RESPONSE TO REVIEWERS’ COMMENTS**

**PGPH-D-24-02493
Development and validation of School Oral Health Promotion Program “SOHEPP” oral health video for the training of adolescents and teachers in Ibadan, Nigeria
PLOS Global Public Health**

We thank the reviewers for the invaluable comments and suggestions, which will make our manuscript richer in quality. Below are the comments and our responses to the comments.

**Editors comment**

The paper provides useful information on the development of a video for oral health education. The manuscript (MS) can benefit from some organization to help readers better follow its flow. Please address the following:

**Comment #1**

**Introduction -** Please revise the MS for English language including mainly grammar such as, for example but not limited to: L64 (an individual journeys)- singular plural mismatch, L72 (oral disease)- this should be in the plural. Please also revise for syntax and flow, like L107 (In addition, the importance of oral to general health has been reported by adolescents in this environment)- this sentence oes fit here and interrupts the flow which was talking about health education. Please, remove it. Please mention the full term for SOHEP the first time it i introduced in Abstract then also again in the MS. Also, divide the text into paragraphs to help readers follow. For example, it may be advisable to begin new paragraphs in Intro L81, L88, and so on

**Response** – Thank you for the comment*.* The manuscript has been edited for grammar incorporating all the suggestions (L74, L84) and that of the English grammar editor.

Also, the sentence “(In addition, the importance of oral to general health has been reported by adolescents in this environment has been deleted (L120 – L121).

The full term of SOHEPP is now mentioned the first time it was introduced in Abstract and in the MS. (L65, L132)

The text has been divided into paragraphs to help readers follow. (L73, L93, L107, L122)

**Comment #2 -** 2- Later (L157), it becomes clear that the 25-minute video is like a story with several scenes (maybe 3). It would be helpful if this is clarified in the Abstract (L47) and earlier in the MS (L117) by using a term to describe the video like story telling or narrative or something similar to allow readers to follow. Until I reached L157, I had no idea whether the video was a lecture, interviews with people with oral health problems or a story.

**Response** *–* Thank you for the comment*.* This has been modified and the video qualified as a narrative (L47, L124).

**Comment #3 -** - Also, the term "animated" is nor very clear. This can be addressed by adding an appendix that includes screenshots of the video to help readers understand. I am not sure if - based on the description of the video in the MS- I would see actors moving and talking in the video or cartoons or other kind of moving characters. Screenshots may also help clarify what is meant by (L197, "illustrations, characters, images, color palette, typography, and keyframes") and what the differences among them are.

**Response -** Screenshots from the videos have been added as Appendices (1 – 3) to ensure clarity on the type of animated characters used for the video.

**Comment #4 -** In addition to the screenshots, it is important to add two separate figures. The first shows the steps of the video development and the second shows the steps of validation. The terms describing the steps in the figures/ illustration should match the subheading in the text elaborating on the steps. In the text, please use numbering to show which steps fit under (1- Development) and (2- Validation). For example, L351 mentions "three versions of the video". What are they? Where do they fit in the development and validation steps?

**Response -** Tables 1, 2 and 3 has been included in the manuscript as suggested. Table 1, illustrating the R&D principles and process of the development, production, and validation of the video. Table 2: Stages in the development and production of the video and Table 3: Process of the validation of the video

**Comment #5 -** Abstract: In Methods, please briefly describe the steps of development and then of validation addressing the how, and by who of both. Please consider adding to the keywords the term "video".

**Response –** The steps of development included pre-video production research, development of the key oral health education messages, development of the script and story boarding, creation, design, and selection of characters, visual style decision, voice-over narration recording, production and post-production validation. This has been included (L50-54).

The produced video was validated for face and content quality among dentists, teachers, and adolescents in secondary schools through individual feedback (one – on- one interviews) and focus group discussions. Relevant comments and observations were considered in the final editing and postproduction process to yield a final video after series of editing.

This has been inserted (L58-62).

Video has been included as a keyword. (L70).

**Comment #6 -** Introduction: Please, support the sentence ending in L93 by a reference.

**Response -** The sentence ending in L93 has been supported by a reference 8-11 (L101).

**Comment #7 -** Methods: L122, please briefly clarify what the R&D model means. Please also in the same line clarify what the principles are. They may be listed afterwards so maybe numbering them in sequence in the paragraph may help readers follow.
**Response** – The R&D model for educational product is a systematic process of investigating the health needs of a target population through research to create new educational products, processes, or technologies and bring them from research stage into production (16).

The ten principles of R&D (Table 1) include Research and Information Collection, Planning, Developing Preliminary Form of Educational Product, Preliminary Field Testing, Revising Main Product, Main Field Testing, Revising Operational Product, Operational Field Testing, Revising Final Product, Disseminating and Implementing the educational product. (L138-145).

**Comment #8** - Methods: Why are 201 adolescents needed for the FGDs? This is a very large number for FG. Saturation must have occurred much earlier than by the time all were included. How many groups were they split into? How long did the meeting with each group take?
**Response** – Thank you. The FGDs was referenced and not discussed in this study. The FGDs involved 201 adolescents because the initial discussions with the students from some schools elicited sparse responses due to fear of dentists from the students as dentists had not previously visited the schools prior to the FGD. Thus, the researchers had to continue with the FGDs until saturation was reached.

This has made the publication of the data obtained from the study as original manuscript difficult due to the scanty transcripts from schools where the adolescents were fearful.

The meetings took 20 to 45 minutes and they were conducted in 18 schools.

**Comment #9 -** Methods L184: Why were revisions needed if back translation showed no differences.

**Response** – Thank you for catching this, there were little differences in some areas of the translation, which required minor corrections like changing articles and none in some areas. The phrase has been deleted.

**Comment #10** - Methods L213: What does "rigging" and "interpolation" mean?

**Response** – Rigging is a process in animation where the skeletal structure that controls the movement of each part of illustration at specific points in time is done. Interpolation is a step during animation creation where smooth movement of the characters is achieved with the aid of some designated software. This has been inserted in (L263-L267).

**Comment #11** - Methods- L232 "responses": Please clarify what the responses were for. Where there close ended questions? a standardized feedback form? or were they asked something like "how did you like the videos"?
**Response** – The responses were from the questions that the teachers were asked on the WhatsApp is as follows: The questions asked after the teachers and students had watched the video included the appropriateness of the video as a training tool for teachers and students, what was good about the video, what should be modified, corrected, removed, or added to it (L295 -L301).
 **Comment #12 -** There is no Results section. Starting L242 "A major comment...." looks like and are Results. Please, put them under the heading of Results.

**Response** – A results heading has been included in the manuscript (L351).

**Comment #13 -** L322-323 "For young children, this cartoon is okay but for adults the human form is the best for them.": How is this music as indicated by the subheading?
**Response** – The music and the character are themes that emerged from the analysis. The two have been separated (L486-L495).

**Comment #14 -** The authors describe the study and data as qualitative. Which qualitative analysis techniques were used for analysis? This needs to be clarified in Methods. Also, in the data availability statement, the authors indicated there is no dataset for this qualitative study. There should be. I assume these are the transcripts that the authors are quoting in the last section of the MS although it is important to clarify how they were obtained and analyzed as indicated in my comment.
**Response** – Thank you for the comment. The transcripts are available upon reasonable request. Details of the analysis and trustworthiness of data have been included (L316 -L349).

**Comment #15** - There is no Discussion section. Qualitative studies and studies developing and validating products do and need Discussion. Please use the STROBE guidelines to develop the Discussion

**Response** – We appreciate the reviewer’s comment, a Discussion section has been added using the STROBE guidelines (L521).

**Reviewer I**

**Comment #1 - Line 36-37:** Presently, in Nigeria, like many other developing countries, oral health promotion programs remain the responsibility of the public health dentist.

**Revise the sentence to:** Presently, in Nigeria, as in many other developing countries, oral health promotion programs remain the responsibility of public health dentists.

**This phrasing removes "the" before "public health dentists," making it clear that the responsibility is generally held by professionals in this field rather than a single individual.**

**Response** – The sentence has been revised as suggested. (Line 38-39)

**Comment #2- Line 47-50:** The video was developed by a team of experts from various specialties, which included public health dentist, pediatric dentist, epidemiologist, children’s illustrator, communication scientist, animation expert, oral and maxillofacial surgery trainee, pediatric surgeon, and science educator.

**If the authors are unaware of the number of individual experts who developed the video, the sentence should be revised to:**

The narrative video was developed by a team of experts from various specialties, which included public health dentists, pediatric dentists, epidemiologists, children’s illustrators, communication scientists, animation experts, oral and maxillofacial surgery trainees, pediatric surgeons, and science educators.

**This version avoids any ambiguity about the number of specialists involved from each field.**

**Response** – The sentence has been revised as suggested.(Line 47-50)

**Comment #3 - Line 64-66:** It would be beneficial to define "adolescent" and state the age range for clarity, especially given that adolescence is central to the study. Defining this term and age range can help readers, particularly those unfamiliar with the exact age group, understand the context and focus of your study.

Adding a definition can be done briefly and naturally, such as: "Oral health conditions and practices vary as an individual journeys through the different stages of life, such as adolescence (1), typically defined as individuals aged 10 to 19 (World Health Organization). This period is characterized by pronounced social and psychological vulnerabilities, which can profoundly impact an individual’s well-being and development (2)."

**Including this information will make the introduction completer and more accessible for readers unfamiliar with specific definitions.**

**Response** – The definition of adolescents has been incorporated as suggested.(Line 76-77).

**Comment #4 - Line 150:** In addition, it also taught skills on effective tooth brushing habits. The right sentence should be “In addition, it also teaches skills on effective tooth brushing habits.”

**Using “teaches” maintains a consistent present tense, which is commonly used when describing the content of educational materials.**

**Response** –The tenses about the video in the paragraph have been changed to present tenses to maintain consistency in the description of the content of educational materials.

“The video portrays basic knowledge of oral health and hygiene, the importance of prevention of oral diseases, as well as the roles of diet, and smoking in oral health. It also illustrates how to recognize symptoms and indicators of poor oral hygiene status such as obvious plaque, calculus, and tooth decay. In addition, it teaches skills on effective tooth brushing habits.” (Line 186 -190).

**Comment #5 - Line 162-165:** He described the sleepless nights he endured, the ineffectiveness of medications and the various harmful substances he had tried such as alcohol, bitter leaf, salt, and “battery water” (water used to refill lead-acid batteries), which only worsened his condition.

**For improving clarity and providing context, here is a revised version of the sentence:**

“He described the sleepless nights he endured, the ineffectiveness of medications, and the various harmful substances he had tried, such as alcohol, bitter leaf (Vernonia amygdalina, a vegetable widely eaten in some parts of Nigeria), salt, and car battery water (water used to refill lead-acid batteries), which only worsened his condition.”

**This version provides additional context about bitter leaf, making it more accessible for readers unfamiliar with it, and clarifies "battery water" to avoid confusion.**

**Response** – Thank you for the suggestion. We have revised the manuscript as suggested (Line 205-211).

**Comment #6 - Line 181-182:** Language experts checked the script for correctness and translated it into Pidgin English and Yoruba.

Change the above sentence to: Language experts checked the script for correctness and translated it into Pidgin English (a widely spoken English-based creole in Nigeria) and Yoruba (a major indigenous language spoken in southwest Nigeria).

**This version makes the sentence more informative and accessible.**

**Response** – Thank you for the suggestion. We have revised the manuscript as suggested to make the sentence more informative and accessible (Line 229-232).

**Comment #7 - Line 252:** You mentioned focus group discussions without an abbreviation, and in line 267 you used the abbreviation “FGD.” Please, make a correction by writing the abbreviation FGD for focus group discussions in line 252**.**

**Response** – Thank you. FGD had been included on Line 166 after focus group discussions. It has been inserted as The FGDs on Line 312.

**Comment #8 - Line 346:** The conclusion should include a policy statement or policy implication. An example of such statement could be **“Given the positive reception of the oral health video by adolescents and teachers, policymakers could consider incorporating such multimedia tools into school curricula as part of a standardized oral health education program.”**

**Response** – This statement has been included in Line 571-573.

**Comment #9 - Line 373: References**

In most academic styles, it is best to avoid mixing capitalization styles within references in a single manuscript. That is what you have in your manuscript. See below:

Lawal FB, Taiwo JO. Making a Case for Formal School-Based Oral Health Promotion: Oral Health Knowledge, Attitude and Practices of Pupils in Ibadan, Nigeria. *Int Q Community Health Educ.* 2018 Jan;38(2):99–105

Popoola BO, Dosumu EB, Ifesanya JU. Periodontal status and treatment need among adolescents in Ibadan, Southwestern Nigeria. *Braz J Oral Sci.* 2015;14(2):117–21

**Using both uppercase and lowercase formats within the same manuscript without a clear rule or pattern may appear unprofessional. To ensure consistency, choose a reference style guide and apply it uniformly across all references in the manuscript.**

**Also, in reference number 15, remove the double quotation marks since other references do not have them.**

**Response** – Thank you for this comment. The suggestions have been incorporated.

**Reviewer #2**

Overall, the paper describes the process undertaken for the development of a training video on dental health aimed at adolescents. It is a qualitative analysis only and limited to 4 dentists, 4 teachers and 4 adolescents each.

**Comment #1** - Attempt could have been made to quantify some of the comments against a prestructured questionnaire.

**Response** – We thank the reviewer for the comment. The R&D model formed the basis of the development and validation of this manuscripts, and it utilizes qualitative methods to explore feedback from the stakeholders, so the results were presented in prose form.

It has been added as a limitation of the study (Line 528-530).

**Comment #2** - Also, the authors do not describe whether there was an attempt made to share the revised video back with those who offered comments.

**Response** *–* The revised video was shared among all those who offered comments until they were. satisfied. This had been included in the manuscript and modified for clarity as,

“Editing and revision of the video was carried out until (there were no more comments from the teachers and adolescents who shared their reservations after watching the video) and were all satisfied.” (Line 517-519).

**Comment #3** - As such the paper describes the normal process of developing such material. No information is available on whether school administrators were also polled for their views on the videos.

**Response** – Thank you. Some of the school administrators were also polled for their views and a quote from one of them was included (the vice principal). This has been clearly state din the manuscript.

“The video was sent to teachers including school administrators such as the vice-principal in four different schools for assessment.” (Line 290-292).

**Comment #4 -** The authors note that the effectiveness of the use of the video in improving dental health and literacy is being assessed. Since the paper is merely describing the standard process that is used for development of such material, the authors fail to convey the value of such a paper. Perhaps it may be a better value to include the assessments that they note as being in progress to be added into the paper to make it more complete.

**Response –** The statement appears to be confusing and has been deleted as this is a methodological study to describe the process, development and validation of a video designed for training students and teachers in the face of sparse number of dentists who have been responsible for the training.

Thank you
