# Supplementary material for: Development and validation of School Oral Health Promotion Program “SOHEPP” oral health video for the training of adolescents and teachers in Ibadan, Nigeria
Source: PLOS Glob Public Health. 2025 Apr 22;5(4):e0004521. doi: 10.1371/journal.pgph.0004521 (PMC12013892; doi:10.1371/journal.pgph.0004521)
Supplement: Attachment — Submitted filename: comments.docx [file pgph.0004521.s007.docx]

REVIWER’S COMMENTS

Line 50: pediatric surgeon should be in plural form

Line 52: story boarding should be storyboarding

Line 53: Full stop is missing after processing

Line 86: There should be a comma after meanwhile

Line 144: story boarding should be storyboarding

Line 147: story boarding should be storyboarding

169: contents should be content

Line 176: story boarding should be storyboarding

Line 177: Contents should be content

Line 178: script writer should be scriptwriter

Line 178: Are you referring to one communication expert or more than one?

Line 259: contents should be content ( **"Content"** (singular) is used when referring to the information, messages, or material within the video as a whole.)

Line 267: Please change **feedbacks** to feedback: Feedback is both singular and collective noun.

Line 269: “if they deemed fit” should be change to “if they deemed it if”

Lines 283-286: The sentence is quite long and complex, which make it difficult for reader to follow. Can you break it down to two sentences? Here is an example of a revised version:

“The FGDs were conducted after the English and Yoruba versions of the video were shown to teachers and students in three different schools, separate from the previously selected schools where the Pidgin version was shown. One FGD was conducted for teachers (as data saturation had been reached), and additional FGDs were held for students. The purpose was to further confirm and triangulate the responses of both teachers and students.”

Line 303: I believe epidemiologist should be in the plural form. Check it out.

Line 304-305: if the moderator is one, then the auxiliary verb (were) should be (was). If not, change the moderator to plural to agree with the verb “were.”

Line 311: The verb ‘has’ should be changed to have.

Lines 476-478: The correct sentence should be: The development of the key messages and content of the video, script, and storyboarding were notable stages, which included experts in oral health, language, and communication arts.

Line 477: Contents should be content

Line 477: story boarding should be storyboarding

Line 491: Please change **feedbacks** to feedback: Feedback is both singular and collective noun.

Linea 495-496: The correct sentence should be: Additionally, reports from around the world highlight the importance of language in the creation of educational materials.

* Do you think it is appropriate to use edutainment as a keyword in the paper? It only appears twice in the text. It's generally recommended that keywords be terms central to your research topic and appear more frequently throughout the text. Keywords should be **significant, relevant, and reflective of the core themes** in your study to improve discoverability in search engines and academic databases. If a term appears only twice but is crucial to your research, you can still use it as a keyword but consider whether other terms might be more representative of your study.
